# Illicit drug use and co-morbidities: a gender perspective

**DOI:** 10.1192/j.eurpsy.2023.130

**Published:** 2023-07-19

**Authors:** M. Torrens

**Affiliations:** ^1^IMIM-Hospital del Mar Research Institut, Barcelona; ^2^Universitat De Vic-Central Catalunya, Vic; ^3^Universitat Autònma Barcelona, Bellaterra, Spain

## Abstract

**Abstract:**

At the global level, women are more likely than men to misuse pharmaceutical drugs, particularly pharmaceutical opioids and tranquillizers. By contrast women are three times less likely than men to use cannabis, cocaine or amphetamines and one in five people who inject drugs are women.This mainly reflects differences in opportunities to use drugs owing to the influence of social or cultural environments, rather than intrinsic gender vulnerability. The scientific literature shows that processes of drug use initiation, social factors and characteristics affecting people who use drugs, biological factors and progression to the development of drug use disorders vary considerably between men and women. In this presentation we will present the main characteristics at bio-psycho-social level, including mental (i.e, Depression, Post- Traumatic Stress disorder) and physical comorbidities (i.e. HIV, HCV) of women with illicit drug use disorders. Finally, there will be a reflection on the different difficulties that women who use drugs have in accessing treatment.

**Disclosure of Interest:**

M. Torrens Consultant of: MT has been a consultant/advisor and/or speaker for Gilead Sciences, Camurus, Servier, Adamed, Lundbeck, Otzuka, Angelini, Molteni

